# An experimental study of VEGF induced changes in vasoactivity in pig retinal arterioles and the influence of an anti-VEGF agent

**DOI:** 10.1186/1471-2415-12-10

**Published:** 2012-07-12

**Authors:** Er-Ning Su, Stephen J Cringle, Ian L McAllister, Dao-Yi Yu

**Affiliations:** 1 Centre for Ophthalmology and Visual Science, The University of Western Australia, Perth, Australia; 2 ARC Centre of Excellence in Vision Science, The University of Western Australia, Perth, Australia

## Abstract

**Background:**

Vascular endothelial growth factor (VEGF) plays an important role in ocular physiology. Anti-VEGF agents are now used for treatment of common retinal diseases. This study characterises the vasoactive properties of VEGF in isolated perfused pig retinal arterioles under normal tone or endothelin-1 (ET-1) pre-contracted conditions and determines the influence of an anti VEGF agent on VEGF induced vasoactivity.

**Methods:**

An isolated perfused retinal arteriole preparation was used. The outer diameter of retinal vessels was monitored at 2 second intervals in response to VEGF and the anti VEGF agent, bevacizumab. The effect of intraluminal delivery of VEGF was determined over a wide concentration range (10^-16^ to 10^-7^ M) both with and without pre-contraction with ET-1 (3 x 10^-9^ M). Bevacizumab (0.35 mg mL^-1^) was applied extraluminally to determine the influence of bevacizumab on VEGF induced vasoactive changes on ET-1 pre-contracted vessels.

**Results:**

In retinal arterioles with normal tone, VEGF induced a concentration dependent contraction at low concentrations, reaching 93.5% at 10^-11^ M and then contraction was reduced at higher concentrations, recovering to 98.1% at 10^-7^ M. VEGF produced a potent concentration dependent vasodilatation in arterioles pre-contracted with ET-1. VEGF induced vasodilatation in arterioles pre-contracted with ET-1 was significantly inhibited by bevacizumab.

**Conclusions:**

VEGF induced vasoactive changes in pig retinal arterioles are dependent on concentration and vascular tone. Bevacizumab inhibits VEGF-induced vasodilatation in pre-contracted arterioles.

## Background

Vascular endothelial growth factor (VEGF) is a protein with a high specificity for endothelial cells. In addition to its role in angiogenesis, VEGF also serves multiple important functions including pro-angiogenesis [1], enhancement of vascular permeability [2], changing vascular tone [3-7], and promotion of cell survival [8], division [9], and differentiation [10].

Neovascular ocular diseases represent a major cause of vision loss in diseases such as proliferative diabetic retinopathy, age-related macular degeneration, retinopathy of prematurity and retinal vascular occlusions [11]. Elevated VEGF has been found in these diseases [12,13]. VEGF has been considered to be an important pathogenic factor as well as a therapeutic target in ocular neovascularisations and associated changes [14]. Given the introduction of therapeutic interventions using VEGF antibodies, VEGF antagonists and VEGF receptor antagonists in clinical ophthalmology, it is more important than ever to understand the normal functions served by VEGF and to understand the consequences of short- and long-term intervention with VEGF inhibitors.

It is critical to address the vasoactive properties of VEGF and anti VEGF agents in retinal vessels, particularly in cases of ischemic ocular diseases. However, little quantitative information is available about the vasoactive properties of VEGF at the retinal arteriole level.

The question addressed in this study is whether VEGF induces direct effects on retinal arterioles and whether it can be influenced by anti-VEGF agents. Our hypotheses are that VEGF can induce concentration dependent effects on retinal arterioles and that these effects can be modulated by anti VEGF agents. In the present study we investigate the vasoactive properties of VEGF in an isolated perfused porcine retinal arteriole preparation. Porcine retinal arteries have been shown to exhibit similar vasoactive properties to human retinal arteries with a range of vasoactive agents [15,16].

## Methods

### Isolated perfused retinal arteriole

Pig eyes were obtained from a local abattoir and picked up by our technician. Following enucleation, the eyes were placed in a sealed bottle of oxygenated Krebs solution and kept on ice during transfer to the laboratory (~60 minutes). All procedures conformed to the EU Directive 2010/63/EU for animal experiments. The dissection, cannulation, perfusion, monitoring and vessel diameter measuring system are fully described in our previous publications using isolated perfused retinal arterioles [15,17-19] and will be only briefly described here.

### Dissection and cannulation of vessels

The eyes were sectioned at pars plana ciliaris, separating the anterior segment and adherent vitreous body from the posterior pole with the aid of a dissecting microscope. The retina, choroid and sclera were divided into quadrants. The retina was then separated from the underlying choroid and sclera. A quadrant of retina was then placed on a hollowed glass slide containing Krebs solution. An individual first-order retinal arteriole was dissected free of retinal tissue with a micropipette. Typically, two arterioles were harvested from each eye. A segment of retinal arteriole (~ 100 μm outer diameter) about 800–1500 μm long and containing only one relatively large side branch was selected. This arterial segment was then relocated to an incubation chamber (PDMI-2, Medical System Corp, New York, USA) mounted on the stage of an inverted microscope (Nikon Diaphot-TMD, Japan). The chamber contained 5 mL Krebs solution. Temperature was maintained at 37^°^C and the incubating solution equilibrated with 95 % O_2_, 5 % CO_2_ so as to maintain PO_2_, PCO_2_ and pH of the incubating solution.

**Figure 1:**
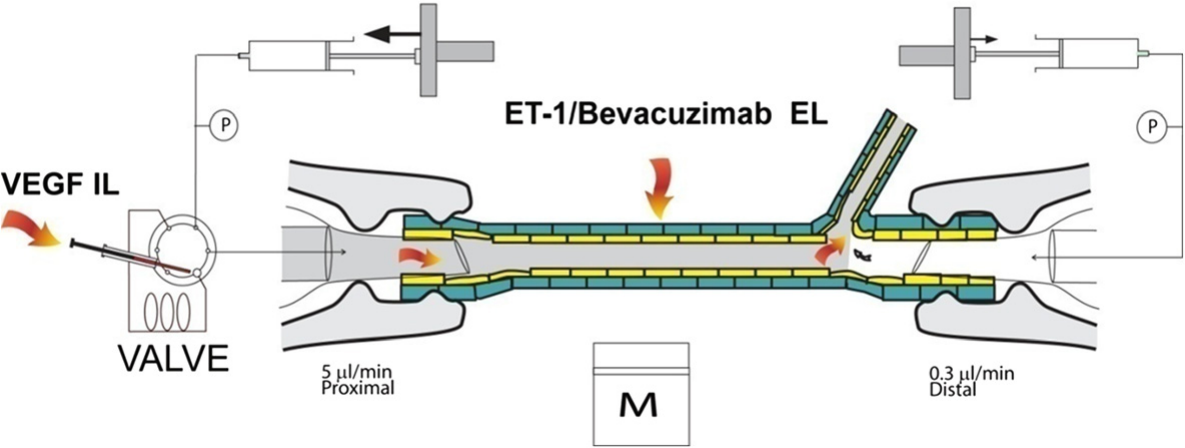
**Schematic representation of the isolated perfused vessel system.** The isolated pig retinal arteriole is cannulated at both ends and perfused intraluminally whilst maintained in a temperature controlled bath on the stage of an inverted microscope. VEGF can be administered intraluminally via an HPLC valve. ET-1 and bevacizumab are delivered extraluminally by addition to the bathing solution. Vessel diameter is monitored every two seconds by an automated frame grabbing and vessel diameter measurement routine.

The arterial segment was then cannulated at both ends using the customized pipette and manipulating system shown schematically in Figure 1. The vessel was then perfused through the proximal end in the orthograde direction at a constant flow of 5 μl min^-1^. The distal end was perfused at 0.3 μl min^-1^ in the retrograde direction to avoid drug entrapment. Both flows exited through the side branch. The vessel was visualized on a video monitor and a pre-programmed computer algorithm was used to measure the external vessel diameter at user selected locations from a frame grabbed image at two second intervals. The vessel was left to stabilize for 30 minutes prior to any drug study. A fresh arteriole was used for each experiment.

### Intraluminal and extraluminal drug delivery

Intraluminal drug delivery was administered as a 5 μl bolus into the perfusate stream *via* an HPLC type sample injector valve. This system allowed the bolus to enter the perfusate stream without pressure artefacts. A switch on the HPLC valve signalled the time of injection of the bolus to the computer and chart recorder. The size, and hence the duration of the bolus, was sufficient for vasoactive responses of the vessel to stabilise. Extraluminal drug delivery was accomplished by direct pipetting into the incubating solution to achieve the required concentration without washing out the bath. The concentration range used of VEGF was 10^-16^- 10^-7^ M for intraluminal delivery. This range covers the concentration range used for VEGF vasoactivity studies in other organs [3,5,6,20,21]. The high cost of VEGF prohibited high concentration levels of VEGF in the relatively large volume of the incubation bath, so only intraluminal VEGF was tested. In a set of preliminary experiments there was no statistically significant difference in VEGF induced vasoactivity in porcine retinal arterioles between intraluminal and extraluminal application (unpublished data). To mimic vitreous injection, bevacizumab was applied extraluminally. All data is presented as normalised vessel diameter percentage, where the data is normalised to the diameter of the vessel prior to any drug administration.

### Solutions and agents

Vessels were usually bathed and perfused with normal Krebs solution of composition (in mM) NaCl 119; KCl 4.6; CaCl_2_ 1.5; MgCl_2_ 1.2; NaHCO_3_ 15; NaH_2_PO_4_ 1.2; Glucose 6. All chemicals and vasoactive agents used were obtained from Sigma Chemicals (St Louis, MO) except recombinant human VEGF which was purchased from Invitrogen, CA and Bevacizumab (Avastin; F. Hoffmann-La Roche, Ltd., Basel, Switzerland) prepared by the Pharmacy Department, Sir Charles Gairdner Hospital, Perth, Australia. The VEGF was dissolved in Na^+^-Krebs solution. Stock solutions were stored at -70^o^C and fresh dilutions were made daily.

### Experiment protocol

After equilibration, an intraluminal injection of 124 mM K^+^ Krebs was given to confirm retinal vessel viability. Vessels were rejected if the contraction response did not result in a diameter of less than 85% of the uncontracted baseline diameter.

The effect of a wide range of intraluminal VEGF concentrations (10^-16^ to 10^-7^ M) was determined in uncontracted retinal arterioles and in arterioles contracted with extraluminal ET-1. When extraluminal application of ET-1 (3 x 10^-9^M) was used to pre-contract the vessels, the ET-1 remained in the bath during all subsequent drug administrations. Pre-contracted vessels can be sustained over the experimental period [19,22,23].

Bevacizumab (0.35 mg mL^-1^) was applied extraluminally after administering extraluminal ET-1 which remained in the bath. This concentration of bevacizumab is comparable to that used clinically [24,25].

### Statistics

All statistical testing was performed using the statistics program SigmaStat (Jandel Scientific Software, San Raphael, CA, USA). The significance of any drug induced concentration dependent changes was tested using one way ANOVA, with significance acceptance level of p < 0.05 for the F value. When comparing concentration response curves, the two way ANOVA using drug concentration as the second factor was used with an acceptance level of P < 0.05. When appropriate, Student’s t test was employed. All mean data is expressed as mean + standard error, and all error bars on graphs are also standard errors. The n number refers to the number of arterioles.

## Results

**Figure 2:**
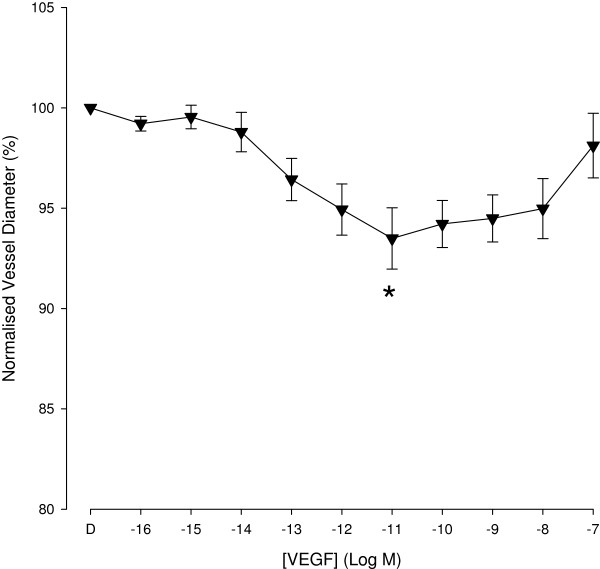
**Concentration response curve of VEGF in retinal arterioles with normal tone.** With intraluminal delivery over a concentration range (10^-16^ – 10^-7^ M) the level of contraction reduced at higher VEGF concentration, producing a significant dilatation when compared to the maximally contracted state at 10^-11^ M VEGF. * Denotes a significant contraction compared to initial baseline (p < 0.05).

The mean baseline diameter of the porcine retinal arterioles prior to any drug administration was 106.4 + 1.4 μm (n = 69). VEGF induced changes in the vessel diameter were not mono-phasic. A wide range of VEFG concentrations (10^-16^ to 10^-7^ M) was administrated to cover this complex change. Average normalised vessel diameter following intraluminal delivery of a range of VEGF concentrations is shown in Figure 2. VEGF produced a significant concentration dependent changes in vessel diameter (p < 0.001). Vessel contraction was maximal at 10^-11^ M VEGF, reaching 93.5 + 1.5 % (n = 24). With higher concentrations, VEGF produced contraction was reduced, reaching only 98.1 + 1.6% at 10^-7^ M.

**Figure 3:**
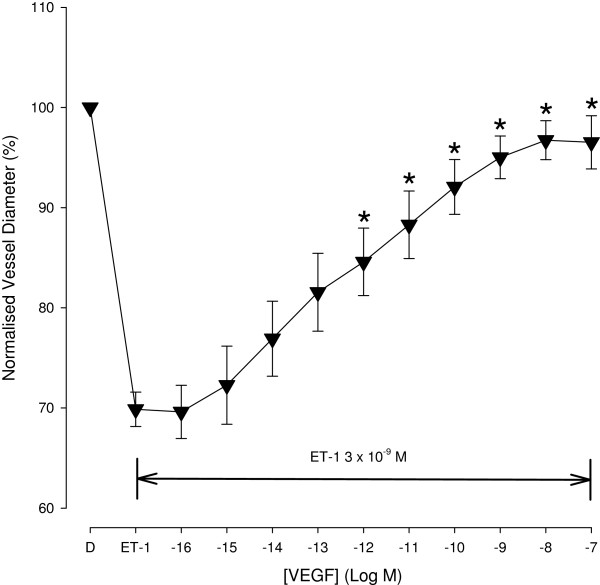
**Concentration response curve of VEGF from ET-1 pre-contracted retinal arterioles.** In isolated pig retinal arterioles pre-contracted with ET-1 (3 x 10^-9^ M) VEGF exhibited a concentration dependent vasodilatation response. * Denotes a significant dilatation compared to contracted baseline (p < 0.05).

However, VEGF induced vasoactivity was altered in prevailing experimental condition. In pre-contracted retinal arterioles, VEGF induced mono-phasic vessel dilatation. ET-1 (3 x 10^-9^ M) was administrated extraluminally, producing an average vessel contraction to 69.9 + 1.7 %. Figure 3 shows the mean normalised diameter change with intraluminal delivery of increasing concentrations of VEGF in ET-1 pre-contracted retinal arterioles. VEGF produced a significant concentration dependent dilatation (p < 0.001). There was a significant dilatation at 10^-12^ M and above when compared with ET-1 pre-contraction alone. The average vessel diameter reached 96.5 + 2.7 % (n = 16) at 10^-7^ M.

**Figure 4:**
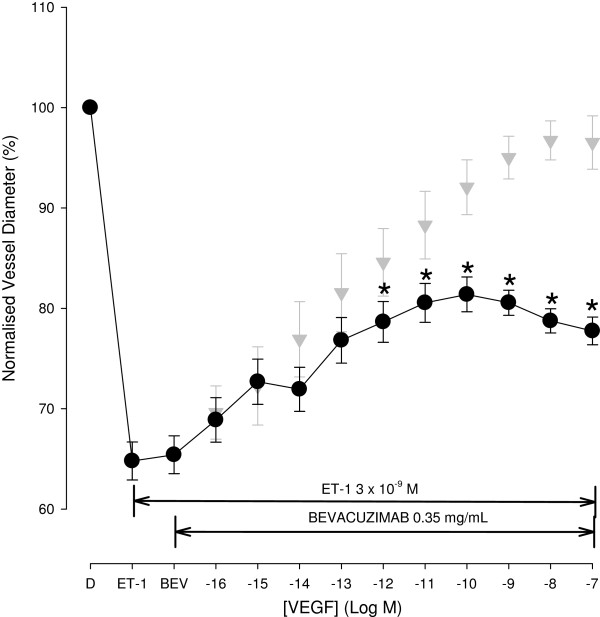
**Concentration response curve of VEGF from ET-1 pre-contracted retinal arterioles with extraluminal bevacizumab.** In isolated pig retinal arterioles pre-contracted with ET-1 (3 x 10^-9^ M) VEGF induced a concentration dependent vasodilation response was significantly inhibited by bevacizumab. For ease of comparison the data showing the VEGF induced dilatation in the absence of bevacizumab is also included (grey symbols). * Denotes a significant dilatation compared to contracted baseline (p < 0.05).

To determine whether bevacizumab can inhibit VEGF induced vasodilatation, a clinical concentration of bevacizumab used for vitreous injection was added extraluminally before VEGF application in ET-1 pre-contracted vessels. Figure 4 shows the mean normalised diameter change with intraluminal delivery of increasing concentrations of VEGF and extraluminal application of both ET-1 (3 x 10^-9^ M) and bevacizumab (0.35 mg mL^-1^). ET-1 administration produced an average vessel contraction to 64.8 + 1.9 % (n = 19) which was not statistically different than that found previously. Adding bevacizumab into the bath did not produce a significant change in vessel diameter (65.4 + 1.9 %, p = 0.815). With the presence of bevacizumab in the bath, VEGF still produced a concentration dependent dilatation (p < 0.001) in ET-1 pre contracted vessels. The averaged vessel diameter was significantly increased at 10^-12^ M and above when compared with that before administration of VEGF. However, the average increase in diameter with VEGF administration was significantly less than that without the addition of bevacizumab (p = 0.002), reaching only 77.7 + 1.4 % at 10^-7^ M. Bevacizumab therefore inhibits VEGF induced vasodilatation responses in pre-contracted vessels.

## Discussion

This study provides detailed information regarding VEGF induced direct vasoactive changes in both normal tone and pre-contracted retinal arterioles and demonstrates that VEGF exhibits vasoactive effects on porcine retinal arterioles. It has been strongly evidenced that the vasoactive properties induced by VEGF in retinal arterioles are clearly depend on the concentration and vessel conditions before the application of VEGF. The results also show that bevacizumab can inhibit the VEGF induced vasodilatations in pre contracted retinal arterioles. These results may be important not only to illuminate and interpret some consequences from patients treated by anti VEGF agents, but also to provide some possible clues as to how to avoid some side effects.

Anti-VEGF agents have been extensively used in ocular diseases in order to inhibit retinal neovascularisation [13,14,26-28].

In considering therapeutic benefits and side effects of VEGF, it is important to bear in mind that VEGF has multiple physiological and pathological roles, and multiple factors could be influenced after modulation of VEGF activity at the systemic or individual organ level. In ocular neovascularisation and ischemic diseases, we need to consider changes in angiogenesis and permeability, and also blood flow. It has been reported that intravitreal ranibizumab could induce retinal circulation disturbances in patients with neovascular age-related macular degeneration [27], neovascular glaucoma [29], retinal vein occlusion [30], and diabetic retinopathy [31]. It is important to determine VEGF induced vasoactivity on retinal arterioles before addressing the possible effects of vitreal bevacizumab injection on the retinal circulation. Our results obtained from retinal arteriole are remarkably different than that from previous reports in other organs. Our results show that the vasoactive properties induced by VEGF in vessels without pre-contraction appear to be mildly contractile at low concentrations (~5% reduction in the vessel diameter) and less contractile at higher concentrations of VEGF (almost reaching the original diameter). However, the results obtained from other organs show dilatation responses of arteries, for example coronary arteries [3,4], placental lobule [32], uterine arteries [20], and pulmonary arteries [21]. One possible explanation is a difference in vasoactive properties between the retinal arteriole and peripheral vessels that were used in most of the previous studies. Retinal blood vessels are not innervated [33-37]. Retinal arteriole tone is mainly regulated by local factors. The mediators derived from the endothelial cells and retinal tissue can be considered as local regulators in addition to physical and metabolic influences [38,39]. However, the effective concentration levels in most previous studies are similar to that in our study indicating that vasoactive effect starts at concentrations as low as ~10^-12^ M [3,6,20,21].

To our knowledge, the effect of VEGF in pre-contracted vessels has not previously been studied. Our results demonstrate that in ET-1 pre-contracted vessels, VEGF induced a potent concentration dependent vasodilatation response which was inhibited by bevacizumab at a similar concentration to that used clinically. The rationale of studying the effect of VEGF on pre-contracted vessels is that the majority of patients treated by bevacizumab are aged, and may have cardiovascular disease and increased vessel tone. Furthermore, VEGF levels in most ocular neovascular diseases are increased. Bevacizumab is a full-length, humanized monoclonal antibody directed against all the biologically active isoforms of vascular endothelial growth factor (VEGF-A). It could be expected that bevacizumab may modulate the retinal vessel tone. Our results demonstrated that bevacizumab can inhibit VEGF induced vasodilatation if the vessel is pre-contracted. Retinal ischemia in some patients may therefore be exacerbated by VEGF inhibition.

Since 2005 the experience with bevacizumab and other anti VEGF agents in ophthalmology has accumulated rapidly. Therapeutic benefits in neovascular ocular diseases have been demonstrated. However, despite the excitement, a number of critical issues have been raised. Two major issues need to be urgently addressed. Firstly, regrowth of new vessels is often found and permanent regression of neovascularization is rare, requiring multiple injections of anti VEGF agent. Secondly, associated ischemia after application of anti VEGF agents has been found in various neovascular ocular diseases. These issues may be interlinked. Neovascularisation in the eye and other organs occurs by both vasculogenesis and angiogenesis. It is now known that vasculogenesis occurs in the adult as endothelial precursor cells derived from the bone marrow enter the circulation in response to ischemic injury. In addition, hypoxia regulated factors are the key mediators of endothelial precursor cells and resident endothelial cells [11]. Ideal treatments of neovascular ocular diseases should not only target reduced neovascularisation and vascular permeability, but also relieve the retinal ischemia/hypoxia if possible. The retina is a functionally active tissue, with arguably the highest oxygen demand tissue per weight, yet the retina has a very limited blood supply from the retinal circulation [40-44]. Recently, reduced retinal perfusion has been demonstrated following anti VEGF treatment in eyes with branch retinal vein occlusion [45]. Anti VEGF treatment further reduces retinal perfusion causing more severe ischemia/hypoxia which could be the cause of the regrowth of new vessels. The potential long-term effects of anti VEGF induced vasoconstriction may need to be considered.

An important question needing to be addressed is how to avoid the associated ischemic retinal and choriocapillaris changes after intraocular administration of anti VEGF agents [27,29-31]. Our results show that bevacizumab is able to inhibit VEGF induced potent vasodilatation. It is possible that the anti-VEGF agent, bevacizumab may inhibit the vasodilatation by VEGF after vitreous injection. As a consequence, retinal blood flow could be reduced. Our results also show that VEGF induced vasodilatation only occurs in ET-1 pre contracted vessels. It could be assumed that in the cases in these clinical reports, VEGF may act as a vasodilator that counterbalances contractile vessel effects caused by the original disease before anti-VEGF injection. However, anti-VEGF agent could inhibit VEGF induced vasodilatation, and as consequence, vasoconstriction could be present. It is well known that VEGF has several family groups but it is the VEGF-A family that is principally involved in the regulation of vasoactive tone. VEGF-A has at least seven splice variants [46]. It could be favourable if an anti-VEGF agent could be chosen that is not involved in the regulation of vasoactive tone, or better still, one that can potentially improve retinal blood flow. The design of a new anti VEGF agent should also consider the differences of vasoactive properties between the retinal vasculature and that in other organs, as well as between normal tone and pre-contracted vessels. In addition, the concentration of anti-VEGF agent also needs to be considered, and case selection may be important. Some cases in which ischemia is predicted, such as ischemic central retinal vein occlusion, added caution in the use of anti-VEGF treatment may be warranted in order to avoid exacerbating retinal ischemia.

There are undoubtedly complex mechanisms regulating ocular and serum levels of VEGF in health and disease. The increasing use of anti-VEGF drugs to treat retinal and choroidal neovascularisation, should be accompanied by further studies of VEGF induced changes in vascular tone in order maximise the therapeutic benefits and to minimise unwanted side effects.

## Conclusions

Using an isolated porcine retinal arteriole preparation we have demonstrated that VEGF causes variable dose dependent effects on arterioles with normal tone, but a potent vasodilatation in arterioles pre-contracted with endothelin-1. The VEGF dilatation effects are significantly counteracted by the anti-VEGF agent bevacizumab. Given the widespread expression of VEGF in tissues and the importance of VEGF for neural cells as well as the endothelium, future detailed studies on biological parameters will be required to elucidate more completely the role of VEGF in the eye.

## Competing interests

The authors declare that they have no competing interests.

## Authors’ contributions

ES performed the experimental work, the initial data analysis, and the initial preparation of the manuscript. DY and SJC designed the study, participated in the experimental work, completed the data analysis, and refined the manuscript. IM assisted with the experiment design and manuscript refinement. All authors read and approved the final manuscript.

## Pre-publication history

The pre-publication history for this paper can be accessed here:

http://www.biomedcentral.com/1471-2415/12/10/prepub
